# Population Structure of Finless Porpoise (*Neophocaena phocaenoides*) Discovered off Coastal Waters, Republic of Korea

**DOI:** 10.3390/genes13101701

**Published:** 2022-09-22

**Authors:** Jeong Eun Ku, Seok-Gwan Choi

**Affiliations:** Cetacean Research Institute, National Institute of Fisheries Science, Ulsan 44780, Korea

**Keywords:** finless porpoise, *Neophocaena phocaenoides*, genetic diversity, population structure, mitochondrial DNA

## Abstract

The finless porpoise (*Neophocaena phocaenoides* Cuvier, 1829) is distributed in the coastal waters of Asia, throughout Indonesia to the east, and as far north as the Taiwan Strait. The finless porpoise has been declared critically endangered by the WWF (World Wide Fund for Nature), and in 2017 was rated vulnerable on the IUCN Red Threatened Species List. Since this species is distributed near the coast and has many interactions with humans, effective conservation of the species requires further studies into their genetic diversity and population. In this study, 45 samples were obtained from bycatch or stranded individuals in the East, South, and West Seas, where Korean porpoises were mainly distributed from 2017–2021. We compared 473 bp mtDNA sequences from the control region. Pairwise fixation indices (*F*_ST_) revealed that the two populations differed significantly (*F*_ST_ = 0.4557, *p* = 0.000). In contrast to high levels of genetic differentiation, gene flow was identified as medium levels (Nm of 0.04–0.71). Our data suggest that finless porpoises may have undergone a historic differentiation event, and that finless porpoises in the three regions could be divided into two populations: West and East/South.

## 1. Introduction

**Figure 1:**
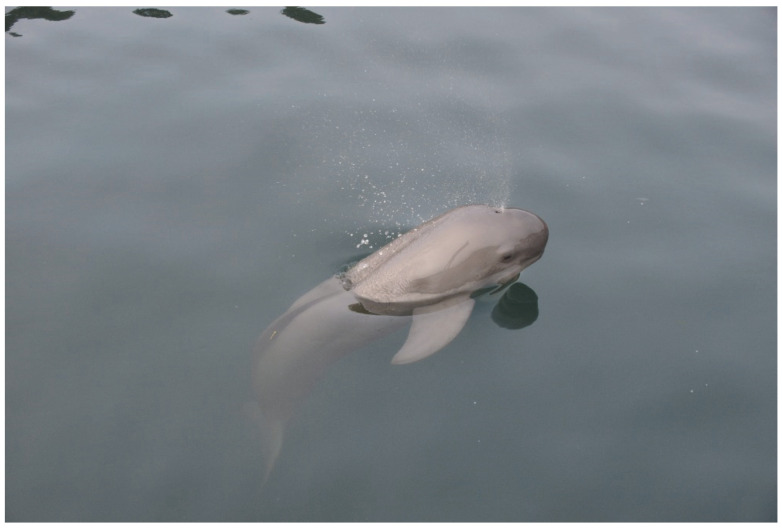
An image of a finless porpoise, *N. phocaenoides*, taken by Hyun Woo Kim.

One of eight porpoise species is the finless porpoise (*N. phocaenoides*, Figure 1), a small, toothed cetacean inhabiting shallow waters along tropical and temperate coastlines [1]. They are also found in the Indo-Pacific coastal waters, from the Persian Gulf to Korea. Although their distribution in Korean waters is known only from Chollabukto (Zenra Hokudo) on the Yellow Sea coast [2], the distribution may be continuous from the Chinese border to the southern coast of the Korean Peninsula [3]. The waters in which they are found are subject to intense human activity, and in some regions, a dramatic reduction in population size has been observed [4]. The porpoise is presented in Appendix l of the Convention on International Trade in Endangered Species (CITES) [1].

Understanding a species’ population genetic structure is necessary for its conservation [5]. Moreover, understanding and maintaining marine ecosystems rely on population genetics [6]. Changes in the biological characteristics and genetic diversity of endangered species may occur when stock management is not based on or is inappropriate for the population structure. As a result, exact population limits for endangered species necessitate a multidisciplinary approach, and genetic studies can provide vital information [7,8]. Although population genetic information is critical for informed management and conservation of endangered species, sampling rare oceanic species remains challenging [6].

Phylogenetic and population genetic investigations have been carried out in order to generate data for the effective management of Phocoenidae species. The finless porpoise’s genetic structure appears to be unusually complex [9]. The majority of studies in China discovered genetic differences in finless porpoises from different geographical locations [10,11,12,13,14,15,16]. Japanese molecular genetics studies using mitochondrial DNA identified five isolated populations in Japanese waters [1,17,18]. Three populations were identified in China [19], and Yoshida et al. [1], studying the structure of five populations off the coast of Japan, proposed that a detailed investigation of the structure of finless porpoise populations across Asia is necessary for the future. In this study, the genetic diversity, gene flow, population structure, and demography of finless porpoises in the East, West, and South Seas off the coast of Korea were compared.

## 2. Materials and Methods

### 2.1. Sample Collection

**Figure 2:**
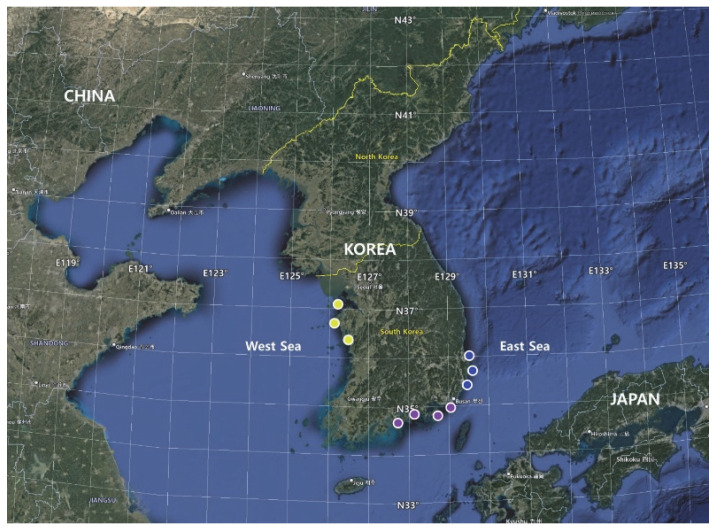
Locations of *N. phocaenoides* samples. E, East Sea (blue circle); S, South Sea (purple circle); W, West Sea (yellow circle).

The finless porpoise is a protected species in Korea. Forty-five finless porpoise muscle tissue samples were collected from bycatch and stranded specimens off the coast of Korea between 2017 and 2021 by a sampler trained by the Korea Coast Guard. The porpoises were collected in the East, South, and West Seas of Korea (Figure 2).

### 2.2. Mitochondrial DNA Sequencing

Genomic DNA was isolated from muscle samples using 10% Chelex 100 Resin (Bio-Rad, Hercules, CA, USA). A 473 bp fragment of the CR was PCR amplified using the following primers: MT4-F (5′-CCT CCC TAA GAC TCA AGG AAG-3′; [20]) and Dlp5-R (5′-GGA TGT CTT ATT TAA GRG GAA-3′; [21]). The PCR reaction mixture had a final volume of 20 μL, including 13.8 μL of distilled water, 2 μL of 10 × PCR buffer, 0.4 μL of 2.5 mM dNTPs, 0.8 μL of each primer, and 0.2 μL of Ex-*Taq* polymerase (TaKaRa, Bio Inc., Shiga, Japan). The thermal cycling included an initial denaturation at 95 °C for 10 min, 29 cycles of denaturation at 94 °C for 30 s, annealing at 50 °C for 30 s, an extension at 72 °C for 1 min, and a final denaturation at 72 °C for 5 min; the mixture was then kept at 4 °C. The PCR products were cleaned using EXOSAP-IT (United States Biochemical Corporation, Cleveland, OH, USA) prior to being sequenced on an ABI 3730XL DNA Analyzer using BigDye Terminator v3.1 Ready Reaction Cycle Sequencing Kits (Applied Biosystems, Foster City, CA, USA). Animal experiments were conducted under the guidance approved by the Animal Research and Ethics Committee of the National Institute of Fisheries Science with the authorization number: 2019-NIFS-11. The newfound mitochondrial haplotypes (control region) were deposited in GenBank, with accession numbers ON470395–ON470439.

### 2.3. Data Analysis

The CR sequences generated were edited using BioEdit ver. 7.2.5 (Informer Technologies, Inc., Roseau Valley, Dominica) [22]. The sequences were aligned using Clustal W [23]. DnaSP ver. 5.10.01 (University of Barcelona, Barcelona, Spain) [24] was used for mtDNA haplotype definition. Fu’s *F*s was used to test for evidence of population expansion [25], while Tajima’s D [26], using DnaSP, was applied for neutrality evaluations for equilibrium in mutational drift. The transitions, transversions, and mismatch distribution parameters were approximated for each specimen through Arlequin ver. 3.5.1.2 (Institute of Ecology and Evolution, University of Bern, Bern, Switzerland) [27]. Nucleotide diversity (*π*; [28]), haplotype diversity (*h*; [29]), and pairwise fixation index (*F*_ST_) were calculated using Arlequin. The median-joining network (MJN) technique [30], using the POPART v.1.7 (University of Otago, Dunedin, New Zealand) [31], was used to infer evolutionary links between haplotypes and haplotype frequencies. In comparison to other rooting and network strategies, such as split decomposition, minimum spanning trees, and TCS, this method has been proven to generate the finest genealogies [32,33]. Gene flow (Nm) was evaluated from pairwise *F*_ST_; that is, Nm = (1/*F*_ST_ − 1)/2 [34].

## 3. Results

### 3.1. Genetic Diversity

We collected 473 bp sequences of the CR from 45 individuals of the finless porpoise population. The CR sequences consisted of 20 haplotypes. Haplotype diversities (*h*) were 1.000 regardless of location; however, nucleotide diversities (*π*) varied by location: 0.003 in the East Sea, 0.004 in the South and West Seas. Analyses of CR variability revealed that the finless porpoise population possessed consistent haplotype and nucleotide diversities, indicating a significant number of distinct haplotypes, as indicated by prior research (Table 1). 

### 3.2. Structure of Population Genetics and Gene Flow

**Figure 3:**
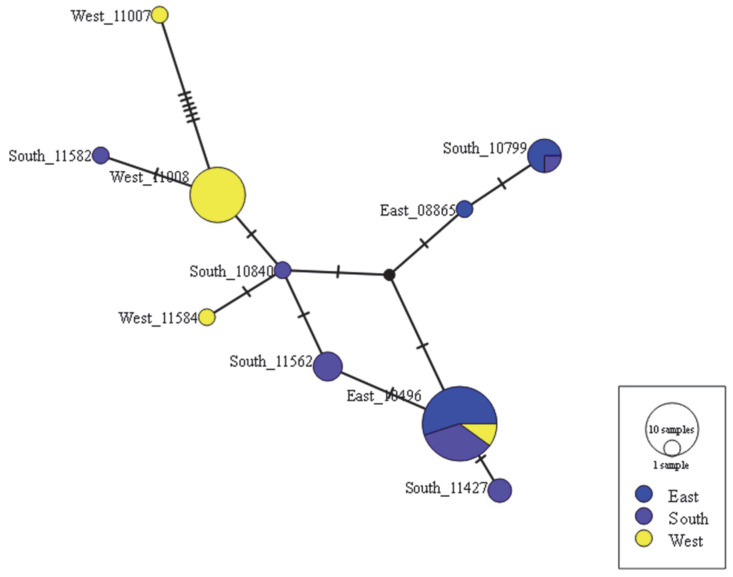
Median joining network-based mtDNA control region sequence haplotypes of 45 finless porpoises, including sequences from East, South, and West Seas. Aligned sequences were 473 bp long. Circle color indicates the region: dark blue, East Sea; purple, South Sea; yellow, West Sea.

In terms of differentiation index, the index of each group in the East, South, and West Seas was 0.374 (*p* < 0.001), and when analyzed with two groups, ES (East and South Seas of finless porpoise; the ES population) and W (West Sea of finless porpoise; the W population), the index was confirmed at 0.456 (*p* = 0.000). The pairwise fixation index (*F*_ST_) was higher in the two populations, W and ES, than in the respective populations of the East Sea, South Sea, and West Sea. This indicated a significant level of genetic differentiation between the two populations, W and ES (Table 2). AMOVA analysis confirmed the genetic variation between the population of 45.57%, and *F*_ST_ showed a significant genetic structure (Table 3). The median joining network of 10 haplotypes revealed that haplotypes are separated into two major clusters (Figure 3). One cluster was identified only as W, whereas the other was constituted of haplotypes obtained from the ES. Moreover, the *F*_ST_ data indicated that a historic differentiation event may have happened between two clusters. Gene flow varied from 0.597 to 1.753 between populations in our study. Following Wright [36], the Nm was categorized into three grades: high (≥1.0), medium (0.250–0.99), and low (0.0–0.249). Our data identified a medium level (Nm = 0.597) gene flow. We found high levels of genetic differentiation (*F*_ST_ of 0.456) and medium levels of gene flow (Nm of 0.04–0.71) between the W and ES populations, based on CR sequences.

### 3.3. Demographic History

The difference between the estimates of *θ*_0_ (0.234) and *θ*_1_ (6827.474) for W was larger than the difference between *θ*_0_ (0.000) and *θ*_1_ (2.246) for ES. The estimates of *θ* for CR showed a larger difference in the W (*θ*_0_ = 0.234, *θ*_1_ = 6827.474) than in the ES (*θ*_0_ = 0.000, *θ*_1_ = 2.246). The W deviated significantly from the neutral constant population size anticipations with statistically significant negative values for Fu’s *F_s_* and Tajima’s *D*. This result reveals a significant excess of singleton mutations relative to the neutral expectation, which is comparable with a bottleneck or a selective sweep [16]. Although Fu’s *F_s_* values of ES showed a significant negative value, Tajima’s *D* showed no significant deviation despite a negative value display. The tau (τ) value of the W was 2.988 and the tau (τ) value of the ES was 1.832, indicating that the estimated time since the sudden population was 87,738 and 53,804 years ago, respectively (Table 4).

## 4. Discussion

Using mtDNA CR sequences, we compared the genetic diversity, population structure, and demographic history of finless porpoises in the East, South, and West Seas. It is believed that the spatial genetic organization of a population is a crucial component in its short-term evolution [37]. It is the outcome of interactions between ecological elements, and it is sensitive to human interventions, historical events, natural selection, and other considerations. The key element impacting the small scale spatial genetic structure of species with a restricted distribution range is limited gene flow. Species with limited gene flow are susceptible to the phenomenon of individual aggregation, which influences the spatial genetic structure of the population [38]. In this investigation, the genetic structure of these populations in the confined space of the East Sea, the South Sea, and the West Sea was determined, and it was observed that the species in this area were divided into two populations, W and ES. It was verified that the medium-level gene flow had an effect on the spatial structure of the population. The genetic diversity of the mtDNA CR sequence of the finless porpoise analyzed here was confirmed to be similar to or slightly higher than that found in previous studies, and the nucleotide diversity was low. These characteristics have also been identified in other cetacean species [39,40,41,42,43]. In the Northwest Atlantic (H: 0.93), the levels of haplotype diversity of CR sequences in the finless porpoise were greater than in the harbor porpoise, *Phocoena phocoena* [39]. The nucleotide diversity of finless porpoises in the present study was lower than that previously reported for finless porpoises in China (*π*: 0.0042) [14]. The mtDNA sequence in this study showed high genetic diversity compared to other Neophocaena species, *Neophocaena asiaeorientalis*. Chen et al. [44] and Zheng et al. [5] reported a nucleotide diversity of 0.0011 for *N. asiaeorientalis* mtDNA CR from another Neophocaena species. The nucleotide diversity (0.0011 [5,44]) and haplotype diversity (0.55 [44],0.65 [5]) were lower compared to the finless dolphins in the present study. High haplotype diversity in conjunction with low nucleotide diversity may indicate recent population expansion, according to a prior study [45]. Recent population growth is consistent with the demographic assessments of the W and ES populations. 

Genetic studies of finless porpoises off the coast of Korea have been conducted previously [46], but no genetic differences were found, due to the limited number of South Sea samples. Yoshida et al. [1] suggested that studies of populations of finless porpoises in all Asian waters are necessary. The current study identified, for the first time, the existence of two distinct clades of finless porpoises in the three seas, a finding which suggests that two distinct populations may be present. An analysis of the pairwise fixation index (*F*_ST_) showed overall significant levels of genetic structuring (*F*_ST_ = 0.4557, *p* = 0.000). As a result of AMOVA analysis, molecular variation was 45.57% higher than in previous studies (41.38% [14]), which is explained by molecular variation between populations, indicating the population structure of the finless porpoise. Using CR markers, Yoshida et al. [1] identified a significant population structure (*F*_ST_ = −0.013–0.893, *p* < 0.05) of finless porpoises in the coastal waters of Japan. These researchers concluded that there was a distinct population in each of the five locations. 

The population structure of finless porpoises was investigated by merging East and South Sea porpoises along the east coast of Korea. Previous research reveals a continuous distribution of porpoises in these waters; therefore, these data have been combined [47,48]. 

Our findings indicate that there is a new demographic structure of finless porpoise off Korean shores. We found a negative and highly significant Tajima’s *D* value, suggesting a historical population growth, a finding which is similar to the results of other studies [15,16]. The Fu’s *Fs* tends to be negative when there is an excess of recent mutations, indicating deviation caused by population expansion and selection [49]. Consistent with the existence of a bottleneck or selective sweep, these data suggest a considerable excess of singleton mutations relative to the neutral expectation [16].

Using a rate of 3.6% per million years for mtDNA CR sequences divergence, the divergence between clusters W and ES was predicted to have occurred 87,738 and 53,804 years ago. During the glacial epoch, a lowered sea level secluded the East Sea from the Pacific Ocean [35,50]. The bearing bottom water appeared during the last glacial age (115,000–11,700 years ago), and the vertical mixing of seawater significantly reduced or stopped during this period [51]. The West Sea (Yellow Sea), located between the Korean Peninsula and mainland China, was entirely exposed during the last glacial period, when the sea level was 120–130 m lower than it is currently [52]. The difference in the values of *θ*_0_ (0.234) and *θ*_1_ (6827.474) is evidence of population expansion in the West Sea, due to the influence of the last glacial age. During this time, there was large-scale iceberg discharge into the Pacific Ocean, and large volumes of freshwater would have been injected into the ocean [53,54]. Therefore, it is possible that major changes have occurred in the Pacific region and in the global climate. The finless porpoise in the East and South Seas may have been affected by the new environment after the large icebergs were ejected. All environmental events affect the status and diversity of genetic populations, which in turn changes genomic regions [55]. This observation supports the possibility of a genetically discrete finless porpoise population in the coastal waters of Korea. In a previous study using a satellite tracking device, Park et al. [56] confirmed that the finless porpoises in the South Sea migrated to the East Sea. 

There was a statistically significant disparity between the West and East populations. Therefore, these populations should be treated as separate management units. Samples from the whole span of the South Sea and the East Sea were not included in this study. Genetic samples of finless porpoises that were bycatch or stranded in the West Sea, the eastern portion of the South Sea, and the southern portion of the East Sea were collected and analyzed. The genetic examination of samples from three marine regions off the coast of Korea, however, provides crucial information for the study of finless porpoises in Asia. In future research, it will be necessary to examine a wide variety of genetic samples from the East, West, and South Seas off the coast of Korea, as well as to examine them in various ways (microsatellite, SNP, NGS, and STR marker-related) by adding samples from the western and northern parts of the South Sea. Our findings indicate the necessity of incorporating Korean population genetics into stock assessment models of finless porpoises in order to ensure their sustainable management. These findings may have substantial consequences for the conservation and management of finless porpoises along the Korean coast.

## Figures and Tables

**Table 1:** Indicators of molecular genetic diversity for the mitochondrial control area of *N. phocaenoides* in Korea.

Location	*n*	*nh*	*H*	*π*	References
East Sea	15	3	1.000 ± 0.0022	0.0031 ± 0.0022	This study
South Sea	15	7	1.000 ± 0.0025	0.0037 ± 0.0025	This study
West Sea	15	10	1.000 ± 0.0029	0.0043 ± 0.0029	This study
SYS	18	10	0.9085 ± 0.0443	0.0042 ± 0.0026	Yang et al. [14]
YS	53	9	0.7138 ± 0.0394	0.0030 ± 0.0022	Yang et al. [14,35]

Abbreviations: *n*, indicates number of individuals; *nh*, number of haplotypes; *h*, haplotype diversity; *π*, nucleotide diversity.

**Table 2:** Mitochondrial genetic differentiation analysis based on mtDNA control region using pairwise fixation index (*F*_ST_) and gene flow (below value) of *N. phocaenoides* samples from coast off Korea.

Location	ESW	SW	EW	ES
ESW	0.374 ** (0.837)			
E		0.222 ** (1.753)		
S			0.071 * (6.511)	
W				0.456 ** (0.597)

* *p* < 0.05, ** *p* < 0.001; Abbreviations: E, East Sea finless porpoise; S, South Sea finless porpoise; W, West Sea finless porpoise; ESW, East, South, and West Sea finless porpoise; SW, South and West Sea finless porpoise; EW, East and West finless porpoise; ES, East and South Sea finless porpoise.

**Table 3:** Analysis of molecular variance (AMOVA) results for the genetic structure of *N. phocaenoides* divided into two groups (ES and W) based on the mtDNA control region sequence.

Source of Variation	Variance	Percentage of Variation	*F*_ST_	*p*-Value
Between populations	0.742	45.57	0.456	<0.001
Within populations	0.887	54.43		

**Table 4:** Summary of the mtDNA sequence variability for populations of *N. phocaenoides*.

Location	*n*	*ti*	*tv*	*π*	Tajima’s *D*	Fu’s *F_S_*	τ	*θ*_0_	*θ*_1_
ES	30	7	0	0.0035	−0.537	−27.486 **	1.832	0.000	2.246
W	15	5	5	0.0043	−1.614 *	−20.081 **	2.988	0.234	6827.474 *
E	15	3	0	0.0031	−0.833	−23.695 **	2.761	0.746	6828.067
S	15	7	0	0.0037	−0.925	−21.762 **	0.633	0.000	26.705

* Significant at *p* < 0.05; ** *p* < 0.001. Abbreviations: *n*, indicates number of individuals; *ti*, number of transitions; *tv*, number of transversions; *π*, nucleotide diversity; τ, *θ*_0_ and *θ*_1_, mismatch distribution parameter estimates from mismatch analyses.

## Data Availability

The data that support the findings of this study are openly available in NCBI at (https://www.ncbi.nlm.nih.gov/, accessed on 20 June 2022), reference number ON470395-ON470439.
